# Mitotane-Induced Hyperlipidemia: A Retrospective Cohort Study

**DOI:** 10.1155/2013/624962

**Published:** 2013-11-14

**Authors:** Hassan Shawa, Ferhat Deniz, Hadil Bazerbashi, Mike Hernandez, Rena Vassilopoulou-Sellin, Camilo Jimenez, Mouhammed Amir Habra

**Affiliations:** ^1^Department of Endocrine Neoplasia and Hormonal Disorders, The University of Texas MD Anderson Cancer Center, Houston, TX 77030, USA; ^2^The University of Texas School of Public Health, The University of Texas Health Science Center at Houston, Houston, TX 77030, USA; ^3^Department of Biostatistics, The University of Texas MD Anderson Cancer Center, Houston, TX 77030, USA

## Abstract

Limited data are available about mitotane-nduced hyperlipidemia. We retrospectively analyzed lipid data in 38 patients with adrenocortical carcinoma (ACC) who received mitotane therapy with emphasis on HDL cholesterol (HDL-c) and clinical predictors of lipid changes. At baseline, the mean levels of HDL-c, LDL-c, and triglycerides were 53.3 mg/dL, 114.4 mg/dL, and 149 mg/dL, respectively. HDL-c, LDL-c, and triglyceride concentrations significantly increased with mitotane therapy to a mean HDL peak (HDL-P) of 86.3 mg/dL (*P* < 0.001), a mean LDL peak of 160.1 mg/dL (*P* < 0.001), and a mean triglyceride peak (Tg-P) of 216.7 mg/dL (*P* = 0.042). HDL-P positively correlated with mitotane concentration (r = 0.52, *P* < 0.001), while LDL-P levels and Tg-P did not. Gender, body mass index, cortisol overproduction, baseline levels of HDL-c, and triglyceride did not predict change in HDL-c. Similar changes were noticed in subgroup analysis after excluding patients who were using lipid-lowering agents. In conclusion, in ACC patients, mitotane caused significant increases in HDL-c that may counteract the deleterious atherosclerotic effects of LDL-c and Tg rise. Understanding the mechanism of HDL change may lead to the discovery of novel HDL-c-elevating drugs.

## 1. Introduction

Mitotane (o,p'-DDD) has been used to treat adrenocortical carcinoma (ACC) for several decades [1]. Mitotane is often given in adjuvant setting after surgical resection of ACC and treatment usually lasts 2-3 years to reduce ACC recurrence. The use of mitotane is associated with multiple adverse effects, including hyperlipidemia. Mitotane increases the levels of low-density lipoprotein cholesterol (LDL-c) via the stimulation of hydroxymethylglutarate-coenzyme A (HMG-CoA) reductase (the rate-limiting enzyme in cholesterol synthesis) [2–7]. Lipid changes in association with mitotane have been reported in small number of subjects. In the largest published report of 17 patients treated with mitotane, high-density lipoprotein cholesterol (HDL-c) increased by 65% from baseline possibly because of the estrogenic effect of mitotane [4]. This HDL-c increase is intriguing as it exceeded what was reported with lipid drugs currently available in clinical practice and remained without clear explanatory mechanism [8, 9]. Preexisting normal triglycerides level was suggested as predictor of mitotane-induced HDL-c increase; however, this was reported in very few patients and was not confirmed in other studies [10].

We conducted a retrospective study in a large cohort of mitotane-treated patients to validate the previously reported observations of mitotane-induced hyperlipidemia, focusing on HDL-c to identify clinical predictors of the mitotane-associated HDL-c changes.Understanding the mechanism of mitotane-induced HDL-c elevation may aid in developing novel drugs that raise HDL-c levels.

## 2. Patients and Methods

This study was approved by the Institutional Review Board of The University of Texas MD Anderson Cancer Center. A search of the institution's database identified 330 patients with ACC treated between 1998 and 2011. Two hundred fifty-three of those patients had received mitotane. We included patients who were 18 years old or older at the time of initiating mitotane. All patients had baseline lipid profiles (before starting mitotane therapy) and at least one additional lipid panel performed at least 30 days after starting mitotane therapy with corresponding mitotane levels. The peak HDL cholesterol (HDL-P) level was defined as the highest HDL-c found during mitotane therapy. As a secondary objective, we also evaluated changes in LDL-c and triglyceride levels with mitotane therapy and recorded the peak LDL cholesterol (LDL-P) and peak triglyceride levels (Tg-P) seen during mitotane use. Obesity, hypertriglyceridemia, and hyperalphalipoproteinemia were defined by a body mass index (BMI) of ≥30 kg/m^2^, a triglyceride level of ≥150 mg/dL, and an HDL-c level of ≥60 mg/dL, respectively. 

Descriptive statistics were used to summarize patient demographic and clinical characteristics. A paired *t*-test was used to assess if the average changes in lipid concentrations from baseline to peak concentration were significantly different from zero. Linear regression was used to assess the relationship between HDL-P, LDL-P, and Tg-P with mitotane concentration while adjusting for patient characteristics of interest. The Pearson correlation coefficient was used to assess the strength of linear relationships between clinical characteristics, and graphical methods were used to visualize relationships of interest. *P* values less than 0.05 were used to assess statistical significance.

## 3. Results

Thirty-eight patients met our inclusion criteria.  Table 1 summarizes the essential characteristics of these patients. The indications for mitotane use included the treatment of metastatic disease (25 patients, 66%), adjuvant therapy after surgical resection (12 patients, 32%), and a treatment for unresectable disease (1 patient, 2%). In the period between mitotane starting date and HDL-P, mitotane was used as a single agent therapy in 28 patients (74%) and combined with chemotherapy in 10 patients (26%) (with etoposide, doxorubicin, and cisplatin in 5 patients, with etoposide and cisplatin in 4 patients, and with streptozocin in 1 patient). At the time of HDL-P, 27 patients (71%) were alive with evidence of ACC recurrence or persistent disease while 11 patients (29%) were reported to be disease free. At baseline, 10 patients were using lipid-lowering agents (7 patients on statins, 1 on omega-3 fatty acids, 1 on ezetimibe, and 1 on both statins and omega-3 fatty acids). At the time of HDL-P, 14 patients were using lipid-lowering agents (10 patients on statins, 2 patients on omega-3 fatty acids, and 2 patients on both). Lipid panels were obtained during a median follow-up time of 292 days (range, 31–1426).

While clinical and biochemical features of Cushing syndrome were reported in 7 patients at the time of initial diagnosis, only 4 patients continued to have evidence of uncontrolled Cushing syndrome at the time of the HDL-P. 

### 3.1. Changes in HDL-c Level with Mitotane

**Figure 1:**
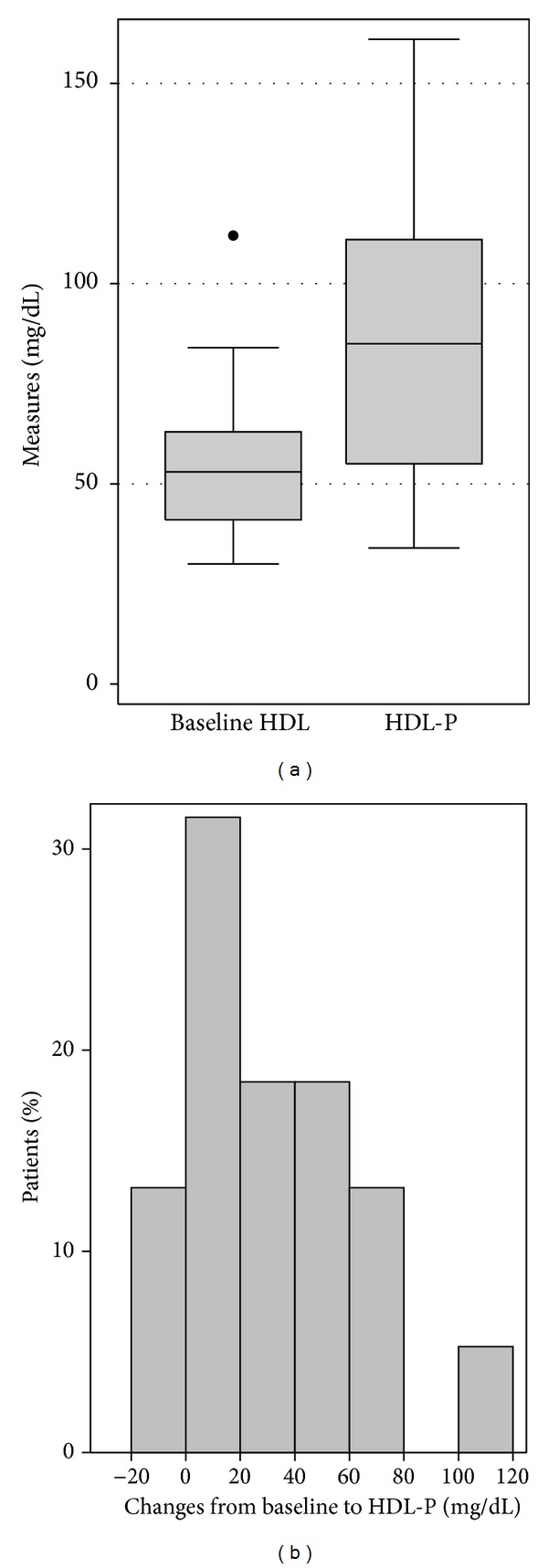
(a) Box plot illustrating HDL-c level at baseline (baseline HDL) and the peak HDL-c during mitotane therapy (HDL-P). Mean baseline HDL was 53.3 mg/dL and mean HDL-P was 86.3 mg/dL (*P* < 0.001). (b) A histogram showing the distribution of within-patient HDL-c changes.

**Figure 2:**
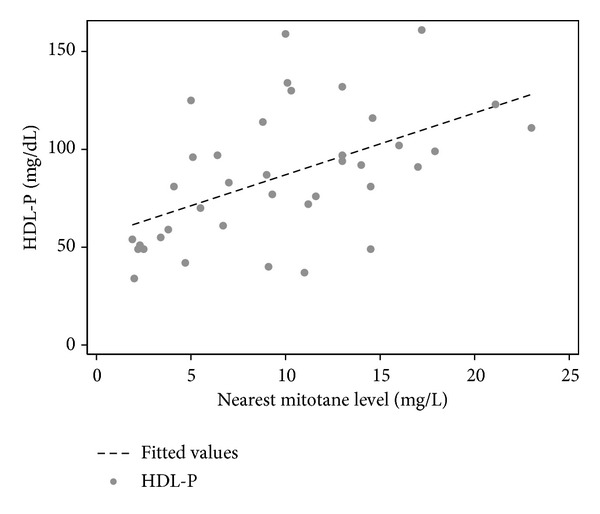
Scatter plot illustrating the relationship between peak HDL-c during mitotane therapy (HDL-P) and mitotane concentrations (*r* = 0.52 and *P* = 0.007) in 38 patients with adrenocortical carcinoma.

The mean baseline HDL-c level was 53.3 mg/dL (±16.3 mg/dL), and HDL-c significantly increased with mitotane therapy to a mean HDL-P of 86.3 mg/dL (±33.6 mg/dL) (mean change = 33.1 mg/dL; *P* < 0.001) (Figure 1). The mean mitotane level (±standard deviation) at the time of HDL-P was 9.8 mg/liter (±5.6 mg/liter). HDL-P and mitotane concentrations showed a significant positive correlation (*r* = 0.52, *P* < 0.001) (Figure 2). The median time for HDL-P was 234.5 days (range, 31–1015 days).

The increase in HDL-c remained statistically significant in a subgroup analysis of the 21 patients who did not receive lipid-lowering agents before or during treatment with mitotane (mean baseline HDL-c : 54.5 mg/dL (±18.6 mg/dL); mean HDL-P : 77.3 mg/dL (±32.9 mg/dL); *P* = 0.009). In addition, the correlation between HDL-P and mitotane concentrations remained statistically significant in that subgroup (*r* = 0.59; *P* = 0.005).

### 3.2. Predictors of HDL-c Change with Mitotane Therapy

At the time of HDL-P, 32 patients had BMI recorded, and the mean BMI was 33 kg/m^2^ (range, 20.5–51.6 kg/m^2^). Twenty-six patients had BMI data both at baseline and at the time of HDL-P. The median BMI change was −0.125 kg/m^2^ (range, −17.1–4.7 kg/m^2^). There was no significant correlation between the change in HDL-c and the change in BMI (*r* = −0.09; *P* = 0.647). The change in HDL-c also did not significantly differ between obese and nonobese patients (*P* = 0.133), men and women (*P* = 0.222), normotriglyceridemic and hypertriglyceridemic patients (*P* = 0.990), patients with cortisol-producing ACC and those with hormonally silent ACC (*P* = 0.530),or in patients with and without hyperalphalipoproteinemia at baseline (*P* = 0.260).

### 3.3. Changes in LDL-c and Triglyceride Levels with Mitotane Therapy

**Figure 3:**
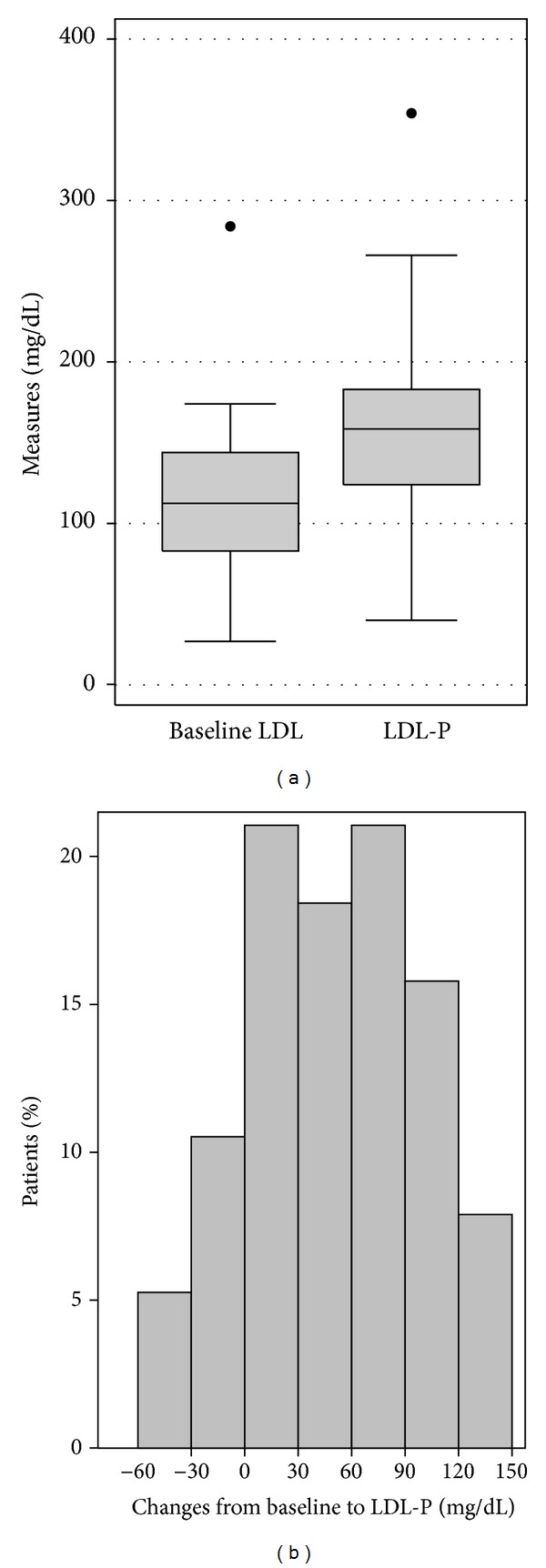
(a) Box plot illustrating LDL-c level at baseline (baseline LDL) and the peak LDL-c during mitotane therapy (LDL-P). Mean LDL-base was 114.4 mg/dL and mean LDL-P was 160.1 mg/dL (*P* < 0.001). (b) A histogram showing the distribution of within-patient LDL-c changes.

The mean baseline LDL-c level was 114.4 mg/dL (±45.5 mg/dL), and LDL-c significantly increased with mitotane therapy to an LDL-P of 160.1 mg/dL (±60.8 mg/dL) (mean change = 45.7 mg/dL; *P* < 0.001) (Figure 3). The median time for LDL-P was 173.5 days (range, 31–1015 days). LDL-P and mitotane concentrations did not correlate significantly (*r* = −0.09, *P* = 0.588). A subgroup analysis of LDL-c changes with mitotane therapy in the 21 patients who were not on lipid-lowering agents revealed a significant increase in LDL-c level (*P* < 0.001), with no significant correlation between LDL-P and mitotane concentrations (*P* = 0.700).

**Figure 4:**
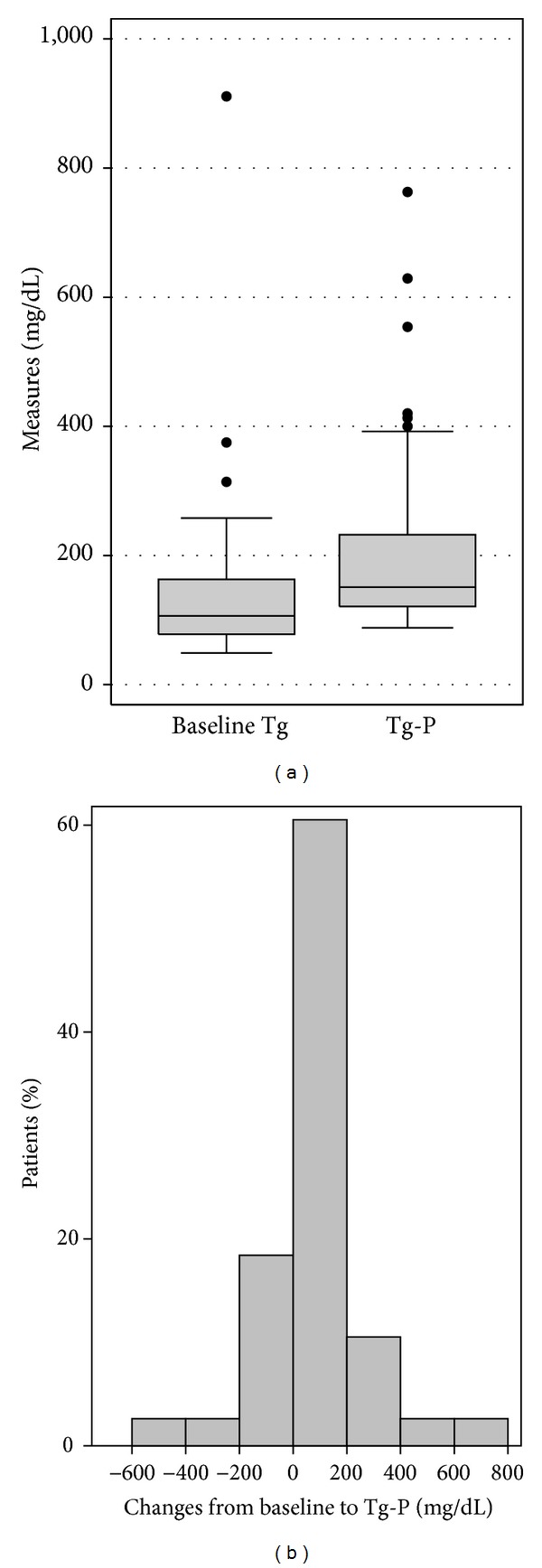
(a) Box plot illustrating triglyceride levels at baseline (baseline Tg) and the peak triglycerides during mitotane therapy (Tg-P). Mean baseline Tg was 149.0 mg/dL and mean Tg-P was 216.7 mg/dL (*P* = 0.042). (b) A histogram showing the distribution of within-patient triglyceride changes.

The mean baseline triglyceride level was 149.0 mg/dL (±146.2 mg/dL), and it significantly changed during mitotane therapy with peak triglyceride of 216.7 mg/dL (±158.6 mg/dL) (mean change = 67.7 mg/dL; *P* = 0.042) (Figure 4). The increase in triglycerides became insignificant after the exclusion of the patients who were on lipid-lowering agents (*P* = 0.056). As with LDL-P, peak triglyceride and mitotane concentrations did not correlate significantly (*r* = 0.02; *P* = 0.908), either with or without inclusion of patients who were using lipid-lowering agents. 

## 4. Discussion

In our cohort, mitotane therapy was associated with a significant increase in HDL-c, LDL-c, and triglyceride levels. Mitotane levels positively correlated with HDL-c but not with LDL-c or triglyceride changes.

Mitotane (o,p'-DDD), an analogue of the insecticide DDT, has been used to treat advanced ACC since the 1960s [1]. Mitotane as adjuvant therapy has been associated with improved ACC outcomes in a large series of patients analyzed retrospectively [11], but it also has multiple adverse effects, which were prospectively assessed and summarized in 17 ACC patients by Daffara et al. [4]. Our work validated the previous observation that mitotane treatment is associated with an increase in HDL-c of about 60–65%, which was directly correlated with serum mitotane concentrations. This increase and correlation were independent of the use of lipid-lowering agents. We did not find any clinical predictors of HDL-c change with mitotane therapy. We also validated the previous reports of a marked increase in LDL-c with mitotane therapy. This increase was independent of the use of lipid-lowering agents and was not correlated with mitotane serum levels. 

HDL-c level is inversely related to the presence or development of coronary heart disease, but a causal relationship between the two has not been well established [12]. Although there is no consistent evidence that raising HDL-c levels is beneficial [13], the risk coronary heart disease has been found to decrease by 2-3% with each increase of 1 mg/dL in HDL-c concentration [14]. Nicotinic acid is the most effective and clinically available HDL-c-increasing therapy, raising HDL-c by 30% when used at a mean dose of 4.5 g/day [8, 15]. However, it is commonly not tolerable by patients owing to various side effects [13]. Cholesteryl ester transfer protein inhibitors represent a new class of lipid-altering drugs that raise HDL-c levels significantly. However, the potential role of these agents remains under investigation in randomized clinical trials.  Table 2 summarizes the predicted change in HDL-c level with each class of lipid-lowering agents. Thus, the HDL-c we have seen in our cohort may have significant cardioprotective effect and may negate the possible deleterious atherosclerotic effect associated with LDL-c increase. It is unclear if lipid-lowering therapy (to treat high LDL-c associated with mitotane) is clinically useful as ACC related death is expected to be more common than cardiovascular death in these patients. In addition, mitotane use is associated with strong CYP3A4 induction that could affect the metabolism of many commonly used statins [16]. 

The mechanism of the HDL-c increase with mitotane therapy is still unclear. The estrogen-like activity of mitotane may be involved; however, the reported increases in HDL-c with estrogen therapy have been only 7–15% and we could not see a difference in HDL-c rise between men and women [20, 21]. It was also hypothesized that mitotane stimulates HDL-c synthesis by inducing the activity of the liver cytochrome P450 [23]. Scavenger receptor class B member 1 (SR-BI) is the primary receptor for the selective uptake of HDL-c and is located mainly in the liver and steroidogenic organs [24]. The carriers of a mutation in SR-BI had increased HDL-c levels without increases in atherosclerosis and had reduced adrenal steroidogenesis [25]. Mitotane is an adrenolytic that commonly leads to mild hepatic impairment [4]. Studies are needed to investigate whether mitotane causes SR-BI dysfunction and whether the corresponding increase in HDL-c is atherogenic. Patients who had bilateral adrenalectomy do not usually develop HDL-c rise [26]. Glucocorticosteroids replacement during mitotane therapy is unlikely to explain HDL-c rise as glucocorticosteroids are often associated with increased plasma levels of total cholesterol, LDL-c, and triglycerides with decreased plasma levels of HDL-c [27]. 

Many adverse events seen during mitotane therapy are dose/level dependent [4]. Thus, it was not surprising that the elevation in HDL-c levels was strongly correlated with increased serum mitotane levels. In contrast, we did not find a significant correlation between LDL-P levels and mitotane concentrations. This observation could be explained by the fact that the increase in LDL-c due to mitotane is independent from mitotane levels; thus, even a low serum mitotane level may be able to stimulate the activity of HMG-CoA reductase strongly enough to significantly increase LDL-c synthesis. 

Obesity, Cushing syndrome, and hypertriglyceridemia are known causes of low HDL-c [28–30], and women have higher HDL-c than men [31]. However, our work revealed that baseline obesity, BMI changes with mitotane therapy, gender, hormonally active ACC, baseline triglyceride levels, and baseline HDL-c levels did not predict HDL-c change with mitotane therapy. Although there was an increase in triglyceride level with mitotane therapy, it was not statistically significant after excluding patients who are on lipid-lowering therapy similar to previous observation by Daffara et al. [4]. 

Our study has the usual limitations of retrospective study design. In particular, we could not determine if blood specimens were collected in fasting or fed state. While this could affect the interpretation triglycerides, it has minimal impact on HDL-c data that are less prone to fluctuation in response to meals [32]. 

Despite these limitations, the size of our cohort is the largest published so far about lipid changes during mitotane use and this allowed us to evaluate multiple clinical predictors and correlate findings to mitotane levels. We showed a marked increase HDL-c with mitotane therapy and a direct, significant correlation between HDL-P and serum mitotane concentrations. The HDL-c increase seems to be greater than the reported experience with the currently available lipid-targeting agents, and it cannot be fully explained by mitotane's estrogenic activity alone. Understanding the mechanism of HDL-c elevation with mitotane could be helpful in developing novel, strong HDL-c raising drugs.

## Figures and Tables

**Table 1:** Demographic and clinical characteristics of the study patients.

Variable	*N* = 38
Mean age at diagnosis, *y* (±standard deviation)	52.5 (±12.5)
Sex, number of patients (%)	
Male	12 (31.6)
Female	26 (68.4)
Race, number of patients (%)	
White	32 (84.2)
Black	3 (7.9)
Hispanic	3 (7.9)
Median baseline body mass index, kg/m^2^ (range)*	34.6 (20.3 to 54)
ACC stage, number of patients (%)	
I	2 (5.3)
II	18 (47.4)
III	13 (34.2)
IV	5 (13.2)
Functional status of ACC, number of patients (%) Functional	15 (39.5)
Hypercortisolism, number of patients (%)	7 (18.4)
Hyperaldosteronism, number of patients (%)	6 (15.8)
Hyperandrogenism, number of patients (%)	2 (5.3)
Nonfunctional, number of patients (%)	23 (60.5)
Mean baseline HDL-c, mg/dL (±standard deviation)	53.3 (±16.3)
Baseline lipid therapy, number of patients (%)	
Yes	10 (26.3)
No	28 (73.7)

*26 patients had information on BMI at baseline.

**Table 2:** Reported HDL-c changes with lipid targeting drugs.

Drug class	Serum HDL-c increase
Bile acid sequestrants	0-1% [17]
Cholesterol absorption inhibitors	1% [18]
HMG-CoA reductase inhibitors	5–10% [9]
Omega-3 fatty acids	5–9% [19]
Estrogen	7–15% [20, 21]
Fibrates	5–20% [8]
Nicotinic acid	15–35% [8, 15]
Fibrates combined with nicotinic acid	up to 45% [15]
Cholesteryl ester transfer protein inhibitors	55–130% [22]
